# Comparison of novel DiaRD-HBV DNA qPCR kit with the *artus* HBV QS-RGQ kit for quantification of HBV DNA in clinical samples

**DOI:** 10.55730/1300-0144.6053

**Published:** 2025-07-08

**Authors:** Ülker ÇUHACI, Hande TOPTAN, Bengül DURMAZ, Mehmet KÖROĞLU, Rıza DURMAZ

**Affiliations:** 1 Department of Medical Microbiology, Faculty of Medicine, Yüksek İhtisas University, Ankara, Turkiye; 2 Department of Medical Microbiology, Faculty of Medicine, Sakarya University, Sakarya, Turkiye; 3 Department of Medical Microbiology, Faculty of Medicine, Lokman Hekim University, Ankara, Turkiye; 2 Department of Medical Microbiology, Faculty of Medicine, Sakarya University, Sakarya, Turkiye; 4 Department of Medical Microbiology, Faculty of Medicine, Yıldırım Beyazıt University, Ankara, Turkiye

**Keywords:** Hepatitis B virus, DiaRD-HBV qPCR kit, *artus* HBV QS-RGQ kit, novel kit

## Abstract

**Background/aim:**

Hepatitis B virus (HBV) leads to widespread chronic infections that may result in long-term complications such as cirrhosis and hepatocellular carcinoma. The use of sensitive molecular methods for HBV DNA quantitation has provided significant contributions for diagnosis and monitoring of disease. The current study aimed to evaluate the diagnostic performances of a novel DiaRD HBV qPCR kit (novel kit) in comparison with the *artus* HBV QS-RGQ kit (comparator kit).

**Materials and methods:**

A total of 288 plasma samples—105 HBV DNA-positive and 183 HBV DNA-negative—were tested using both kits. The diagnostic and quantitative results of the novel kit were compared to those of the comparator kit.

**Results:**

The novel kit had very high sensitivity, specificity, and accuracy with rates of >99.9%. Comparative analyses conducted on 105 HBV DNA-positive samples showed no significant differences of ≥ 1 log_10_ between the novel kit and comparator kit. The median viral load detected by the novel kit was 6.21 × 10^7^ IU/mL (range: 0.9 × 10^1^ to 2.98 × 10^9^) as compared to the comparator kit where it was 5.43 × 10^7^ IU/mL (range: 1.00 × 10^1^ to 2.87 × 10^9^). The correlation plot demonstrated a strong linear relationship (R^2^ value of 0.9655) between the two kits. Reproducibility testing performed on three different HVB DNA loads showed the overall coefficient of variations <5%. The mean difference between the quantitative results of two kits was −0.03 log IU/mL by Bland–Altman analysis.

**Conclusion:**

The result of the current study indicates that this novel DiaRD HBV qPCR kit can be used in routine diagnosis and monitoring of HBV infections.

## 1. Introduction

Hepatitis B virus (HBV) is a small, enveloped DNA virus in the *Hepadnaviridae* family [1]. HBV replicates in the liver cells of humans and leads to an acute or chronic infection. Chronic hepatitis B (CHB) virus infection that might result in cirrhosis and liver cancer is a well-known threat to global health [2,3].

Occult hepatitis B infection is another subcategory that is characterized with detectable or undetectable HBV DNA in the plasma of patients testing negative for the HBV surface antigen [4,5].

Hepatitis B is a major health problem worldwide. WHO estimated that 254 million people were living with chronic hepatitis B infection in 2022, with 1.2 million new infections each year. In 2022, hepatitis B led to an estimated 1.1 million deaths, mostly from cirrhosis and hepatocellular carcinoma.^1^ HBV infection is a significant health problem in Türkiye. The estimated number of HBV carriers in Türkiye is approximately 3.3 million and the overall HBV prevalence is 4.57% [3].

HBV can be controlled by means of diagnosis and effective treatment. However, based on WHO estimates, as of 2022, only 13% of people with CHB infection can be diagnosed and nearly 3% of them were able to receive antiviral therapy at the end of 2022.^2^ It is not possible to clinically distinguish hepatitis B from hepatitis caused by the other viral agents. Therefore, laboratory confirmation of the diagnosis is important. The most reliable test used to determine HBV DNA quantitation in the plasma of hepatitis B patients is the real time polymerase chain reaction (Rt-PCR) method [6,7]. The DiaRD-HBV qPCR kit (Diagen Inc, Ankara/Türkiye) is a quantitative Rt-PCR that has all the necessary components to quantify HBV DNA in plasma sample of hepatitis B patients. The kit consists of primers for amplification of the 90-base pair region in the HBV S gene and a fluorophore dye and quencher-labeled TaqMan probe to detect amplification. The kit has plasmid-derived internal control DNA with the same length and similar base content as the target gene. It features a primer binding site identical to that of the target gene, but with a probe binding site unique to the internal control. The kit includes five quantitation standards for determination of HBV DNA load, as well as positive and negative controls.^3^ The aim of the study was to evaluate the clinical performance of a novel qPCR kit, the DiaRD-HBV qPCR kit, by comparing it with the *artus* HBV QS-RGQ kit (QIAGEN GmbH, Germany) and assessing the clinical utility of DiaRD-HBV kit in hepatitis B diagnosis.

## 2. Materials and methods

### 2.1. Ethical approval

Ethical approval was obtained from Sakarya University, Medical Faculty Ethical Committee (Ethical Committee number: E-71522473-050.01.04-230880-89 and date of approval: 15.03.2023).

### 2.2. Clinical samples

A total of 105 HBV DNA-positive and 183 HBV DNA-negative plasma samples submitted to Medical Microbiology Laboratory of Sakarya University Education and Research Hospital during 2023 for routine quantitative HBV DNA testing by *artus* HBV QS-RGQ kit were retrospectively selected to evaluate performance of the DiaRD-HBV qPCR kit. Blood samples in standard sample collection tubes containing EDTA were centrifuged to separate plasma within the first 6 h after collection. The plasma samples were then stored at −70 °C after the testing by the comparator kit. Before testing with the novel kit, patient information was anonymized. No specific information such as the patient’s name, surname, ID number, or phone number was used.

### 2.3. DNA isolation

The DiaRD HBV qPCR kit was validated for DNA samples extracted from plasma using the Diarex Viral DNA/RNA extraction kit protocol (Cat. No: VDR-8786, Diagen Inc, Ankara/Türkiye www.diagen.com.tr). Briefly, 200 μL of plasma was mixed with 25 μL of proteinase K, 300 μL of lysis buffer, and 5 μL of internal control. After a quick mixing and incubation at 56 °C for 5 min, 300 μL of absolute ethanol was added and vortexed. The entire lysate was transferred into a spin column and centrifuged at 8000 × *g* for 1 min. The spin column was placed back into the collection tube and washed with wash buffer-1 and wash buffer-2. In the last step, the DNA in spin column was eluted with 30 μL of elution buffer. For the comparator kit, DNA purification was performed on the QIAsymphony SP using a QIAsymphony DSP Virus/Pathogen Kit by strictly following the kit’s instructions.^4^

### 2.4. RT-PCR protocols

All DNA samples were processed using both novel and comparator kits, following the respective kit protocols. Total qPCR reaction volume and amount of target DNA for the comparator kit were 50 mL and 20 mL, respectively. These volumes for the novel kit were 20 mL and 5 mL, respectively. An amplification program for the novel kit was constructed with the first denaturation at 95 °C for 10 min, following 45 cycles including denaturation at 95 °C for 10 s and annealing and extension at 55 °C for 50 s. The comparator kit has an initial denaturation step at 95 °C for 10 min following 45 cycles including 95 °C for 15 s, 55 °C for 30 s, and 72 °C for 15 s. While DNA extraction and reaction setup were manually performed in the novel kit, in the comparator kit, these processes were performed by automated systems following the kit’s instructions.^5^

### 2.5. Reproducibility of the novel kit

In accordance with CLSI EP05-A3 recommendations, reproducibility studies of the novel kit were performed with 9.55×10E+03, 9.55x10E+02, and 9.55×10E+01 IU/mL dilutions of the reference material (4th WHO International Standard for HBV DNA for NAT, NIBSC code: 10/266) in five replicates per day during five days.^6^ The Ct and HBV DNA loads data were used to estimate the standard deviation, the variance, and the coefficient of variation for the novel kit.

### 2.6. Statistical analysis

SPSS Software v. 17 was used for statistical analysis. Sensitivity, specificity, and overall agreement were calculated to compare concordance between two kits in positive and negative clinical samples. The correlation analysis of HBV DNA viral load values obtained in the dynamic measurement range of both kits was performed and the correlation coefficient “R” value was calculated. In addition, the quantitative results, expressed in log_10_ IU/mL and obtained within the dynamic measurement range of both kits, were analyzed using Bland–Altman analysis. The scatter of their differences against the mean was examined. Deming regression analysis was performed to investigate the degree of agreement between the results of both kits. The DATAtab web application for data analysis was used to perform Bland–Altman and Deming regression analyses.^7^

## 3. Results

All 105 plasma samples identified as positive by the comparator kit were also positive with the DiaRD-HBV qPCR kit (novel kit). Positive agreement between the two kits was 100%. All 183 HBV-negative samples yielded negative qPCR results with both kits. When results obtained from the comparator kit were used as reference, the specificity and sensitivity of the novel kit were estimated as >99.9%. The overall agreement between the two kits was 100% (Table 1).

**Figure 1:**
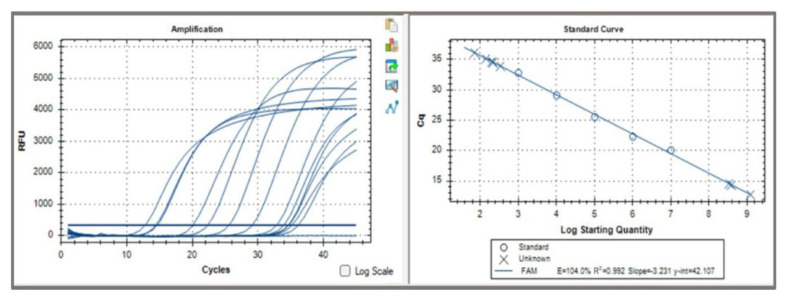
An example of the novel qPCR results on representative clinical samples and quantitation standards.

qPCR studies performed with the novel kit had efficiency between 90 and 110, RFU values of >2000, and R2 value of the quantitation standards ranging from 0.955 to 1.00 (Figure 1).

The median viral load values measured by the novel kit and the comparator kit were calculated as 6.21 × 10^7^ IU/mL (range: 0.9 × 10^1^ to 2.98 × 10^9^) and 5.43 × 10^7^ IU/mL (range: 1.00 × 10^1^ to 2.87 × 10^9^), respectively. When the results were analyzed in log_10_ IU/mL, the median viral load value was 3.39 log_10_ IU/mL (range: 0.95 to 9.47) for the novel kit and 3.36 log_10_ IU/mL (range: 1 to 9.46) for the comparator kit. Among the 105 samples, 97 (92.4%) had a measurement difference of ≤±0.5 log_10_ IU/mL, and eight (7.3%) had a difference of >±0.5 log_10_ (range: 0.675 to 0.83 log_10_). These eight samples had DNA concentrations between 5.8 × 10^1^ and 1.62 × 10^4^ IU/mL (mean: 2.98 × 10^3^ IU/mL). Of these eight samples, five had DNA concentrations between 58 and 566 IU/mL, two had DNA levels of 1160 IU/mL and 5400 IU/mL, and one had a DNA level of 16,200 IU/mL. The sample with HBV DNA value of 16,200 IU/mL with the comparator kit had a low DNA level (3450 IU/mL) with the novel kit. Four of the eight discordant samples had higher HBV DNA concentrations detected by the novel kit than those detected by the comparator kit, and the remaining four had lower HBV DNA levels detected by the novel kit than those detected by the comparator kit. There was no sample having a significant difference of ≥±1 log_10_ IU/mL (Table 2).

As shown in Table 3, a comparison of the results of the novel kit and comparator kit by means of SD and % coefficient of variation (CV) as per log values provided additional data to support the presence of good correlation between the two kits (Table 3).

**Figure 2:**
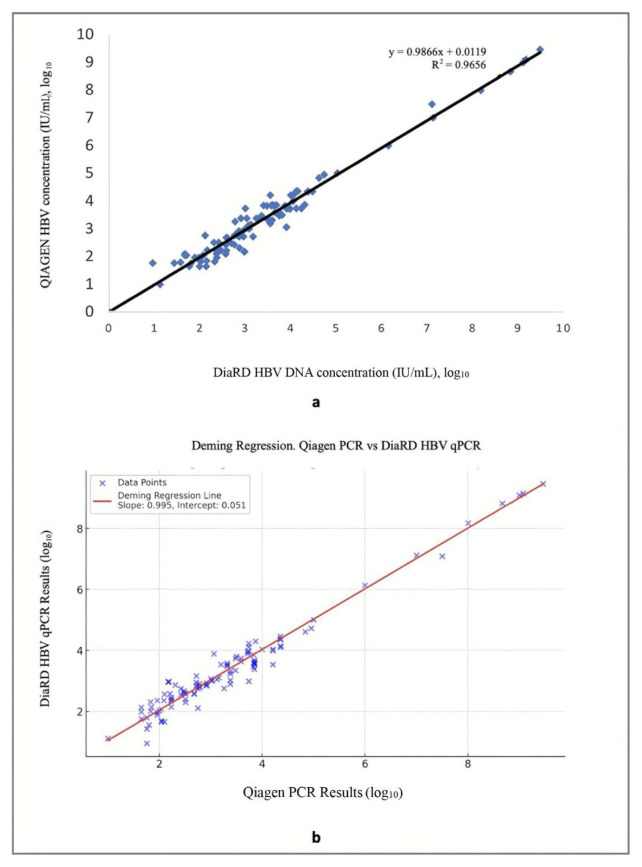
Correlation between quantitative results of DiaRD HBV qPCR kit and Artus HBV QS-RGQ kit (QIAGEN GmbH, Germany). The R^2^ values for linear (2a) and Deming (2b) regression analysis were 0.966 and 0.995, respectively.

The quantitative results obtained from positive samples with both kits were compared by linear and Deming regression analyses to investigate the degree of agreement of the results. There was a high concordance (R ^2^ ≥ 0.966) between the quantitative results of both tests (Figures 2a and 2b).

**Figure 3:**
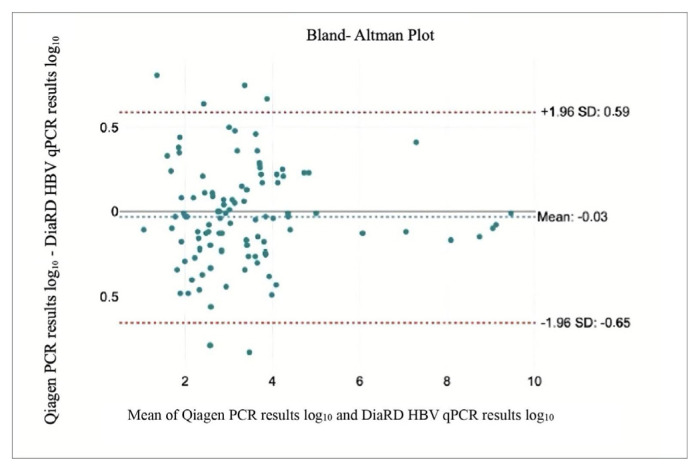
Bland–Altman analysis result showing the mean log difference (−0.03), upper limit of agreement (+1.96 SD: 0.59) and lower limit of agreement (1.96 SD: −0.65).

The difference between the HBV viral load log_10_ IU/mL values obtained from 105 samples by both kits was plotted against the average of the log_10_ IU/mL results of the kits tested. Bland–Altman test results showed that the difference between both tests was within the limit of agreement for 99 of the 105 samples (95% confidence interval, CI). The lowest difference between the comparator kit and the novel kit value was −0.81, the highest difference was 0.83, and the mean difference was −0.03 [standard deviation (SD) = 0.31] IU/mL with a 95% CI. The comparator kit resulted in slightly lower measurements than the novel kit (Figure 3).

In the reproducibility study, according to the results of the statistical analysis performed with the Ct values obtained, the overall CV was found to be between 0.47% and 0.60%. Concerning HBV DNA loads, the overall CV values ranged from 2% to 4.9%. The intraassay CV rates from five different experiments ranged from 8.49% to 18.9% for the sample with a DNA load of 9.55 × 10^3^ IU/mL, 19.4% to 23% for the sample with a DNA load of 9.55 × 10^2^, and 57% to 78.6% for the sample with a DNA load of 9.55 × 10^1^ IU/mL (Table 4).

## 4. Discussion

Quantification of HBV DNA in plasma samples has a crucial impact on the decision to initiate antiviral treatment and monitor the response to treatment. Currently, there are several qPCR kits having various clinical performances and many companies have developed alternative kits for these purposes [8–12]. The newly developed qPCR kits have been compared with comparator kits before they were widely used in routine diagnosis [10–12,13]. The current study presents first data regarding clinical performance of the DiaRD HBV qPCR kit, which is a novel quantitative kit having an analytic sensitivity of 19.8 IU/mL and a linearity range of 3.5–1.295 × 10^9^ IU/mL, and which is able to detect all HBV genotypes (A to J).^8^ We found that the novel kit has quite a high performance, having 100% sensitivity, specificity, and accuracy. Comparative analyses demonstrated no differences of ≥±1 log_10_ between the novel and comparator kits. Moreover, the differences >±0.5 log_10_ (ranging from 0.67 to 0.81 log_10_) were observed in only eight samples. In the regression analysis, the obtained R^2^ values of 0.966 and 0.995 indicate that more than 96% of the measurements obtained in both kits are comparable to each other. Besides, the slope of the linear regression equation (y = 0.9868) indicates that the measurement values of the novel kit are nearly the same as those of the comparator kit.

It is well known that qPCR assays have high sensitivity, specificity, broad dynamic range, and are able to accurately quantitate HBV DNA in clinical samples. A recent study carried out on clinical samples to compare performances of a new Amplisure HBV Quantitative assay with the *artus* QS-RGQ assay showed a positive agreement of 100% with an overall agreement of 94%, and good correlation (R^2^ = 0.967) between the two kits [8]. Another study conducted on 118 clinical samples to compare performances of RTA RT-PCR kit and *artus* RG RT-PCR for detection of HBV DNA in plasma samples found a high correlation between the two kits (R^2^ = 0.955) [14]. Recently, the performance characteristics of a TRUPCR (3B Black Bio Biotech, India Ltd., India) were compared with those of the *artus* HBV kit (Qiagen, Germany) in 121 HBV-infected patients. It was reported that there was a high correlation (R^2^ = 0.964) and an overall concordance of 92.6% between the two assays [15]. Another study comparing droplet digital PCR with Abbot’s real-time PCR in 54 clinical samples reported a correlation coefficient of 0.788 and a diagnostic sensitivity of 96.3% [11]. Deming regression of HBV DNA levels measured by the Alinity m HBV assay and Xpert HBV viral load assay in 91 plasma specimens showed a strong correlation (R^2^ = 0.982; y = 0.934 × 1 0.22) [16]. In that study, the overall agreement between two systems was reported as ≥96.7%. Parallel to these results, the DiaRD HBV qPCR kit had 100% sensitivity, specificity, and accuracy rates, and an R^2^ value of ≥0.966, indicating that the novel qPCR kit can be an alternative for available qPCR systems.

To investigate the degree of closeness of the results of two kits, Bland–Altman analysis was performed by drawing a scatter plot of the differences against the means of the measurements obtained from the two tests. Accordingly, while the novel kit measured slightly higher HBV DNA loads than the comparator kit, the mean difference with the comparator kit was only 0.03 log_10_ IU/mL. This result is below 0.5 log_10_ IU/mL, which indicates a biologically significant viral load increase in PCR tests. Beside high correlation (R^2^ ≥ 0.966), 0.03 log_10_ difference obtained by Bland–Altman analysis provided additional data for utility of the DiaRD HBV qPCR kit in clinical samples. In agreement with our results, several studies have also reported low deviations between the quantitative values of the qPCR systems. Like our result, in a previous study, Bland–Altman analysis performed on the results of the Artus HBV QS-RGQ Assay and Amplisure HBV DNA kit found a very low difference (0.09 IU/mL) between two tests [9]. In another study, mean difference between RTA-RT PCR kit and *artus* RG RT-PCR was found to be 0.4 log IU/mL by Bland–Altman analysis [14]. A slight deviation (0.03±0.31 log IU/mL) was also reported between the quantitative results of the Alinity m HBV assay and the Xpert HBV viral load assay [17]. Bland–Altman plot analysis of the Aptima HBV Quant assay versus the Abbott RealTime HBV assay detected a deviation of 0.24 log_10_ [18]. These results showed that the measurement outcomes of the DiaRD-HBV kit are highly consistent with those of the comparator kit and other systems compared in the literature.

In a reproducibility test, we detected low rates of variation with an overall CV value of ≤4.9%. Coefficient of variation values obtained from reproducibility tests are at a similar level to existing commercial kits used in quantitation of HBV DNA. According to the literature, coefficient of variation values below 15% are generally considered acceptable [16]. In a recent study, both interassay and intraassay variations obtained by the TRUPCR kit were reported as CV of <5% [15]. A current study reported 0.29% to 5.07% intraassay and 4.90% to 6.85% interassay coefficients of variation for Aptima HBV Quant assay [17]. In the COBAS TaqMan HBV test, according to the manufacturer’s information, it was stated that the intraassay variation ranged from 7% to 50%, and interassay variation ranged from 11% to 26% according to DNA loads tested.^9^ In *artus* HBV QS-RGQ kit, these values were reported as 9.28% and 7.92%, respectively.^10^ Previous studies indicated that low HBV DNA levels and/or genotype variations lead to discordance results between kits [8,9,11,14]. It was reported that HBV genotype B led to significant discrepancy in HBV DNA viral load detection [18]. Although we did not perform HBV genotyping in the current study, previous data showed that almost all HBV viruses in Türkiye are genotype D [19]. In agreement with the statement indicating that low HBV DNA viral loads result in discrepancy, of the eight samples showing discordance results in our study, five had HBV DNA load less than 5.66 × 10^2^ IU/mL, two had HBV DNA as 1.16 × 10^3^ IU/mL and 5.44 × 10^3^ IU/mL, and one had HBV DNA concentration of 1.62 × 10^4^ IU/mL. Similarly, in another study, the rate of discordant results obtained by a novel kit and comparator test was high in the samples having HBV DNA levels less than 4 × 10^1^ IU/mL [15]. Conversely, a previous study indicated that the highest concordance between the qPCR and droplet digital PCR (ddPCR) results was observed for the detection of samples with the lowest copy numbers (<10^3^ copies/μL) of HBV DNA; however, when the copy number of HBV DNA was higher than 10^6^ copies, the ddPCR droplets result in false quantification [11]. As was indicated previously [10], the variations detected in low DNA loads may be related to the level of analytical sensitivity and/or linearity of the kits. The DiaRD HBV kit’s 95% LOD of 19.8 IU/is about two-fold above than that of the comparator kit (10.2 IU/mL) and the linear range of the novel kit (3.5–1.295 × 10^9^ IU/mL) is better than that (31.6 to >2 × 10^7^ IU/mL) of the comparator kit.^11^ Thus, careful consideration should be given to test selection, especially in cases of low viral loads.

The consistent quantitative results obtained with the comparator and the novel kit, along with the high sensitivity and specificity for the DiaRD-HBV kit, are important indicators for diagnostic reliability of the novel kit. The high analytic sensitivity of the novel kit indicates the ability to detect low viral loads, while the high specificity value indicates minimal risk of misidentifying individuals without HBV infection as false positives. Thus, the high diagnostic accuracy of the DiaRD-HBV kit is critically significant, particularly for diagnosing and directing treatment for individuals at high risk. However, there are some limitations to this study. First, the sample size and diversity of the study population are insufficient to support recommending the kit for use in the general population. Although the developer of the DiaRD HBV kit claims it can detect all HBV genotypes, it is important to assess the consistency of its performance across different genotypes using clinical samples. Furthermore, since the study was conducted using samples from a single hospital, additional studies involving diverse populations are needed to evaluate the kit’s performance under real-world conditions.

In conclusion, this is the first study showing that the newly developed DiaRD HBV qPCR assay is sensitive, specific, and reproducible, and accurately quantifies HBV DNA levels in plasma samples. Its performance is consistent with commercial IVD licensed kits commonly used for detection and quantification of HBV loads in clinical practice. Furthermore, the novel kit is an open system that can be used in any molecular laboratory which has a Bio-Rad and Rotor Gene thermal cycler. It can be a good alternative for commercial kits in a routine diagnostic laboratory.

## Figures and Tables

**Table 1:** Comparison of DiaRD HBV qPCR kit with the *artus* HBV QS-RGQ kit.

	*artus* HBV QS-RGQ kit		

**DiaRD qPCR kit**	Positive	Negative	Total
**Positive**	105	-	105
**Negative**	-	183	183
**Total**	105	183	288

**Sensitivity (%)**	100	-	-
**Specificity (%)**	-	100	-
**Overall agreement (%)**	-	-	100

**Table 2:** Quantitative results of the 105 HBV DNA-positive samples tested by DiaRD qPCR kit and Qiagen *artus* HBV QS-RGQ kit.

Sample no.	Qiagen PCR results (IU/mL)	DiaRD HBV qPCR results (IU/mL)	Mean (IU/mL)	SD (IU/mL)	Qiagen PCR results (log_10_)	DiaRD HBV qPCR results (log_10_)	Mean (log10)	SD (log_10_)	Difference between log_10_ values
**1**	2.87×10^9^	2.98×10^9^	2.93×10^9^	5.50×10^7^	9.46	9.47	9.465	0.005	0.01
**2**	6.11×10^2^	6.18×10^2^	6.15×10^2^	3.50	2.79	2.79	2.79	0	0
**3**	1.24×10^2^	2.28×10^2^	1.76×10^2^	5.20×10^1^	2.09	2.36	2.225	0.135	0.27
**4**	3.13×10^7^	1.24×10^7^	2.19×10^7^	9.45×10^6^	7.5	7.09	7.295	0.205	−0.41
**5**	8.27×10^2^	8.60×10^2^	8.44×10^2^	1.65×10^1^	2.92	2.93	2.925	0.005	0.01
**6**	4.50×10^1^	1.35×10^2^	9×10^1^	4.50×10^1^	1.65	2.13	1.89	0.24	0.48
**7**	6.30×10^1^	3.66×10^1^	4.98×10^1^	1.32×10^1^	1.8	1.56	1.68.	0.12	−0.24
**8**	5.99×10^2^	6.62×10^2^	6.31×10^2^	3.15×10^1^	2.78	2.82	2.8	0.02	0.04
**9**	5.66×10^2^	5.61×10^2^	5.64×10^2^	2.50	2.75	2.75	2.75	0	0
**10**	5.80×10^1^	0.9×10^1^	3.35×10^1^	2.45×10^1^	1.76	0.95	1.355	0.405	−0.81
**11**	4.50×10^1^	9.71×10^1^	7.11×10^1^	2.61×10^1^	1.65	1.99	1.82	0.17	0.34
**12**	3.89×10^3^	5.54×10^3^	4.72×10^3^	8.25×10^2^	3.59	3.74	3.665	0.075	0.15
**13**	4.74×10^2^	3.75×10^2^	4.25×10^2^	4.95×10^1^	2.68	2.57	2.625	0.055	−0.11
**14**	1.68×10^2^	2.21×10^2^	1.95×10^2^	2.65×10^1^	2.23	2.35	2.29	0.06	0.12
**15**	1.22×10^9^	1.46×10^9^	1.34×10^9^	1.20×10^8^	9.08	9.16	9.12	0.04	0.08
**16**	5.70×10^1^	6.19×10^1^	5.95×10^1^	2.45	1.76	1.79	1.775	0.015	0.03
**17**	3.23×10^2^	1.98×10^2^	2.61×10^2^	6.25×10^1^	2.51	2.3	2.405	0.105	−0.21
**18**	2.08×10^3^	3.11×10^3^	2.60×10^3^	5.15×10^2^	3.32	3.49	3.405	0.085	0.17
**19**	6.70×10^1^	1.01×10^2^	8.40×10^1^	1.70×10^1^	1.83	2.01	1.92	0.09	0.18
**20**	7.11×10^3^	3.93×10^3^	5.52×10^3^	1.59×10^3^	3.85	3.59	3.72	0.13	−0.26
**21**	1.68×10^2^	2.84×10^2^	2.26×10^2^	5.80×10^1^	2.23	2.45	2.34	0.11	0.22
**22**	5.44×10^3^	9.86×10^2^	3.21×10^3^	2.23×10^3^	3.74	2.99	3.365	0.375	−0.75
**23**	1.10×10^2^	4.92×10^1^	7.96×10^1^	3.04×10^1^	2.04	1.69	1.865	0.175	−0.35
**24**	3.23×10^2^	3.85×10^2^	3.54×10^2^	3.10×10^1^	2.51	2.59	2.55	0.04	0.08
**25**	9.20×10^1^	7.57×10^1^	8.39×10^1^	8.15	1.96	1.88	1.92	0.04	−0.08
**26**	4.72×10^8^	6.54×10^8^	5.63×10^8^	9.10×10^7^	8.67	8.82	8.745	0.075	0.15
**27**	6.70×10^1^	2.04×10^2^	1.36×10^2^	6.85×10^1^	1.83	2.31	2.07	0.24	0.48
**28**	7.11×10^3^	3.72×10^3^	5.42×10^3^	1.70×10^3^	3.85	3.57	3.71	0.14	−0.28
**29**	5.26×10^2^	7.14×10^2^	6.20×10^2^	9.40×10^1^	2.72	2.85	2.785	0.065	0.13
**30**	2.28×10^4^	1.29×10^4^	1.79×10^4^	4.95×10^3^	4.36	4.11	4.235	0.125	−0.25
**31**	3.23×10^2^	2.51×10^2^	2.87×10^2^	3.60×10^1^	2.51	2.4	2.455	0.055	−0.11
**32**	9.20×10^1^	9.25×10^1^	9.23×10^1^	2.50×10^1^	1.96	1.97	1.965	0.005	0.01
**33**	3.05×10^2^	4.83×10^2^	3.94×10^2^	8.90×10^1^	2.48	2.68	2.58	0.1	0.2
**34**	8.27×10^2^	7.00×10^2^	7.64×10^2^	6.35×10^1^	2.92	2.85	2.885	0.035	−0.07
**35**	5.26×10^2^	8.85×10^2^	7.06×10^2^	1.80×10^2^	2.72	2.95	2.835	0.115	0.23
**36**	8.84×10^4^	5.27×10^4^	7.06×10^4^	1.79×10^4^	4.95	4.72	4.835	0.115	−0.23
**37**	2.28×10^4^	1.40×10^4^	1.84×10^4^	4.40×10^3^	4.36	4.15	4.255	0.105	−0.21
**38**	1.10×10^2^	1.16×10^2^	1.13×10^2^	3.00	2.04	2.07	2.055	0.015	0.03
**39**	1.51×10^2^	9.27×10^2^	5.39×10^2^	3.88×10^2^	2.18	2.97	2.575	0.395	0.79
**40**	9.20×10^1^	2.32×10^2^	1.62×10^2^	7.00×10^1^	1.96	2.36	2.16	0.2	0.4
**41**	5.24×10^3^	9.13×10^3^	7.19×10^3^	1.95×10^3^	3.72	3.96	3.84	0.12	0.24
**42**	1.25×10^2^	4.59×10^1^	8.55×10^1^	3.96×10^1^	2.1	1.66	1.88	0.22	−0.44
**43**	2.39×10^3^	1.06×10^3^	1.73×10^3^	6.65×10^2^	3.38	3.02	3.2	0.18	−0.36
**44**	2.41×10^3^	2.11×10^3^	2.26×10^3^	1.50×10^2^	3.38	3.32	3.35	0.03	−0.06
**45**	1.10×10^2^	4.54×10^1^	7.77×10^1^	3.23×10^1^	2.04	1.66	1.85	0.19	−0.38
**46**	1.51×10^2^	9.41×10^2^	5.46×10^2^	3.95×10^2^	2.18	2.97	2.575	0.395	0.79
**47**	1.62×10^4^	3.45×10^3^	9.83×10^3^	6.38×10^3^	4.21	3.54	3.875	0.335	−0.67
**48**	2.64×10^2^	3.57×10^2^	3.11×10^2^	4.65×10^1^	2.42	2.55	2.485	0.065	0.13
**49**	5.24×10^3^	8.02×10^3^	6.63×10^3^	1.39×10^3^	3.72	3.9	3.81	0.09	0.18
**50**	2.39×10^3^	7.91×10^2^	1.59×10^3^	8.00×10^2^	3.38	2.9	3.14	0.24	−0.48
**51**	2.41×10^3^	1.71×10^3^	2.06×10^3^	3.50×10^2^	3.38	3.23	3.305	0.075	−0.15
**52**	5.44×10^3^	1.30×10^4^	9.22×10^3^	3.78×10^3^	3.74	4.12	3.93	0.19	0.38
**53**	1.51×10^2^	9.26×10^2^	5.39×10^2^	3.88×10^2^	2.18	2.97	2.575	0.395	0.79
**54**	2.64×10^2^	5.63×10^2^	4.14×10^2^	1.50×10^2^	2.42	2.75	2.585	0.165	0.33
**55**	3.01×10^3^	5.49×10^3^	4.25×10^3^	1.24×10^3^	3.48	3.74	3.61	0.13	0.26
**56**	7.05×10^3^	3.66×10^3^	5.36×10^3^	1.70×10^3^	3.85	3.56	3.705	0.145	−0.29
**57**	1.62×10^4^	1.09×10^4^	1.36×10^4^	2.65×10^3^	4.21	4.04	4.125	0.085	−0.17
**58**	2.22×10^4^	2.38×10^4^	2.30 ×10^4^	8.00×10^2^	4.35	4.38	4.365	0.015	0.03
**59**	5.26×10^2^	1.44×10^3^	9.83×10^2^	4.57×10^2^	2.72	3.16	2.94	0.22	0.44
**60**	5.44×10^3^	1.68×10^4^	1.11×10^4^	5.68×10^3^	3.74	4.23	3.985	0.245	0.49
**61**	2.08×10^3^	3.28×10^3^	2.68×10^3^	6.00×10^2^	3.32	3.52	3.42	0.1	0.2
**62**	1.05×10^3^	1.01×10^3^	1.03×10^3^	2.00×10^1^	3.02	3.01	3.015	0.005	−0.01
**63**	1.68×10^2^	1.41×10^2^	1.55×10^2^	1.35×10^1^	2.23	2.15	2.19	0.04	−0.08
**64**	2.22×10^4^	2.32×10^4^	2.27×10^4^	5.00×10^2^	4.35	4.36	4.355	0.005	0.01
**65**	2.22×10^4^	2.92×10^4^	2.57×10^4^	3.50×10^3^	4.35	4.46	4.405	0.055	0.11
**66**	7.44×10^3^	1.98×10^4^	1.36×10^4^	6.18×10^3^	3.87	4.3	4.085	0.215	0.43
**67**	2.08×10^3^	3.77×10^3^	2.93×10^3^	8.45×10^2^	3.32	3.58	3.45	0.13	0.26
**68**	7.05×10^3^	4.28×10^3^	5.67×10^3^	1.39×10^3^	3.85	3.63	3.74	0.11	−0.22
**69**	8.06×10^2^	7.37×10^2^	7.72×10^2^	3.45×10^1^	2.91	2.87	2.89	0.02	−0.04
**70**	6.84×10^4^	4.08×10^4^	5.46×10^4^	1.38×10^4^	4.84	4.61	4.725	0.115	−0.23
**71**	5.80×10^1^	2.66×10^1^	4.23×10^1^	1.57×10^1^	1.76	1.43	1.595	0.165	−0.33
**72**	1.44×10^3^	1.29×10^3^	1.37×10^3^	7.50×10^1^	3.16	3.11	3.135	0.025	−0.05
**73**	3.89×10^3^	4.37×10^3^	4.13×10^3^	2.40×10^2^	3.59	3.64	3.615	0.025	0.05
**74**	1.58×10^3^	3.45×10^3^	2.52×10^3^	9.35×10^2^	3.2	3.54	3.37	0.17	0.34
**75**	6.75×10^3^	2.94×10^3^	4.85×10^3^	1.91×10^3^	3.83	3.47	3.65	0.18	−0.36
**76**	7.10×10^1^	1.37×10^2^	1.04×10^2^	3.30×10^1^	1.85	2.14	1.995	0.145	0.29
**77**	1.83×10^3^	5.80×10^2^	1.21×10^3^	6.25×10^2^	3.26	2.76	3.01	0.25	−0.5
**78**	6.75×10^3^	7.27×10^3^	7.01×10^3^	2.60×10^2^	3.83	3.86	3.845	0.015	0.03
**79**	1.16×10^3^	7.78×10^3^	4.47×10^3^	3.31×10^3^	3.06	3.89	3.475	0.415	0.83
**80**	1.25×10^2^	3.61×10^2^	2.43×10^2^	1.18×10^2^	2.1	2.56	2.33	0.23	0.46
**81**	4.50×10^1^	5.69×10^1^	5.10×10^1^	5.95	1.65	1.75	1.7	0.05	0.1
**82**	3.05×10^2^	3.96×10^2^	3.51×10^2^	4.55×10^1^	2.48	2.6	2.54	0.06	0.12
**83**	1.05×10^3^	1.02×10^3^	1.04×10^3^	1.50×10^1^	3.02	3.01	3.015	0.005	−0.01
**84**	7.05×10^3^	2.46×10^3^	4.76×10^3^	2.30×10^3^	3.85	3.39	3.62	0.23	−0.46
**85**	1.64×10^2^	3.76×10^2^	2.70×10^2^	1.06×10^2^	2.21	2.58	2.395	0.185	0.37
**86**	2.06×10^2^	7.34×10^2^	4.70×10^2^	2.64×10^2^	2.31	2.87	2.59	0.28	0.56
**87**	1.31×10^3^	1.13×10^3^	1.22×10^3^	9.00×10^1^	3.12	3.05	3.085	0.035	−0.07
**88**	4.74×10^2^	3.90×10^2^	4.32×10^2^	4.20×10^1^	2.68	2.59	2.635	0.045	−0.09
**89**	1.68×10^2^	2.46×10^2^	2.07×10^2^	3.90×10^1^	2.23	2.39	2.31	0.08	0.16
**90**	5.66×10^2^	1.28×10^2^	3.47×10^2^	2.19×10^2^	2.75	2.11	2.43	0.32	−0.64
**91**	5.24×10^3^	9.35×10^3^	7.30×10^3^	2.06×10^3^	3.72	3.97	3.845	0.125	0.25
**92**	7.11×10^3^	4.78×10^3^	5.95×10^3^	1.17×10^3^	3.85	3.68	3.765	0.085	−0.17
**93**	5.99×10^2^	8.06×10^2^	7.03×10^2^	1.04×10^2^	2.78	2.91	2.845	0.065	0.13
**94**	1.62×10^4^	9.87×10^3^	1.30×10^4^	3.17×10^3^	4.21	3.99	4.1	0.11	−0.22
**95**	3.01×10^3^	2.22×10^3^	2.62×10^3^	3.95×10^2^	3.48	3.35	3.415	0.065	−0.13
**96**	3.14×10^3^	6.32×10^3^	4.73×10^3^	1.59×10^3^	3.5	3.8	3.65	0.15	0.3
**97**	1.00×10^9^	1.25×10^9^	1.13×10^9^	1.25×10^8^	9	9.1	9.05	0.05	0.1
**98**	1.00×10^8^	1.46×10^8^	1.23×10^8^	2.30×10^7^	8	8.17	8.085	0.085	0.17
**99**	1.00×10^7^	1.32×10^7^	1.16×10^7^	1.60×10^6^	7	7.12	7.06	0.06	0.12
**100**	1.00×10^6^	1.36×10^6^	1.18×10^6^	1.80×10^5^	6	6.13	6.065	0.065	0.13
**101**	1.00×10^5^	1.03×10^5^	1.02×10^5^	1.50×10^3^	5	5.01	5.005	0.005	0.01
**102**	1.00×10^4^	1.10×10^4^	1.05×10^4^	5.00×10^2^	4	4.04	4.02	0.02	0.04
**103**	1.00×10^3^	1.18×10^3^	1.09×10^3^	9.00×10^1^	3	3.07	3.035	0.035	0.07
**104**	1.00×10^2^	1.08×10^2^	1.04×10^2^	4.00	2	2.03	2.015	0.015	0.03
**105**	1.00×10^1^	1.29×10^1^	1.15×10^1^	1.45	1	1.11	1.055	0.055	0.11

**Table 3:** Comparison of the results of DiaRD HBV qPCR kit and comparator kit by means of SD and percentage of CV as per log values.

Log values	Qiagen *artus* comparator kit				DiaRD HBV qPCR kit			

	n	Mean	SD	% CV	n	Mean	SD	%CV
**<2**	14	1.74	0.23	13.2	12	1.62	0.30	18.5
**2**	36	2.44	0.28	11.4	40	2.57	0.30	11.6
**3**	35	3.51	0.28	7.97	32	3.51	0.30	8.54
**4**	11	4.38	0.26	5.93	12	4.29	0.21	4.89
**>5**	9	7.74	1.43	18.4	9	7.78	1.45	18.6

**Table 4:** Intra- and interassay variation and reproducibility of the novel kit.

HBV DNA load	First day		Second day		Third day		Fourth day		Fifth day		Overall	
9.55 × 10^3^ IU/mL	Ct value	IU/mL	Ct value	IU/mL	Ct value	IU/mL	Ct value	IU/mL	Ct value	IU/mL	Ct value	IU/mL
Mean	30.5	4004	30.5	4004	30.5	4430	30.6	4360	31.0	4333	30.6	4230
Standard variation	0.12	339.97	0.12	339.97	0.34	761	0.42	483.84	0.37	819	0.14	206.5
Coefficient of variation (CV) %	0.40	8.49	0.40	8.49	1.11	17.2	1.39	11.10	1.19	18.9	0.47	4.9
9.55 × 10^2^ IU/mL												
Mean	33.4	457	33.4	457	33.60	470	33.70	621.80	33.56	442	30.62	457
Standard variation	0.3	88.5	0.3	88.48	0.49	71.2	0.65	242.88	0.43	102	0.14	10.3
Coefficient of variation (CV) %	0.8	19.4	0.8	19.36	1.45	15.2	1.94	39.06	1.29	23	0.47	2.3
9.55 × 10^1^ IU/mL												
Mean	36.5	61.02	36.52	61.02	37.04	61	36.7	58.93	36.94	58.68	36.74	60.1
Standard variation	1.3	47.95	1.26	47.95	0.92	37.15	0.83	34.05	0.79	34.99	0.24	1.2
Coefficient of variation (CV) %	3.5	78.6	3.5	78.6	2.5	60.9	2.3	57.8	2.1	59.6	0.70	2.0

## Footnotes

1 World Health Organization (2024). Global hepatitis report 2024: action for access in low- and middle-income countries (online), https://www.who.int/publications/i/item/9789240091672 [accessed 19 February 2025]

2 World Health Organization (2024). Global hepatitis report 2024: action for access in low- and middle-income countries (online), https://www.who.int/publications/i/item/9789240091672 [accessed 19 February 2025]

3 DiaGen. Hepatitis B Quantitive Rt-PCR kit (online), https://www.diagen.com.tr/hepatitis-b-quantitive-rt-pcr-kit [accessed 19 February 2025]Top of FormBottom of Form

4 Qiagen. QIAsymphony® DSP Virus/Pathogen Kit Instructions for Use (Handbook), file:///C:/Users/10006402/Downloads/HB-3028-001_HB_QS_DSP_VirusPathogen_Kit_IVDR_0622_WW_1127539%20(2).pdf [accessed on 19 February 2025]

5 Qiagen. artus® HBV QS-RGQ Kit Handbook (online), file:///C:/Users/10006402/Downloads/HB-0335-004_1060925_148048620_R4_HB_artus-HBV-QS-RGQ-CE_1214_ROW.pdf [accessed on 19 February 2025]

6 Clinical Laboratory Standard Institute. Evaluation of precision quantitative measurement procedures; approved guideline-3rd ed. (online), https://clsi.org/standards/products/method-evaluation/documents/ep05/ [accessed on 19 February 2025]

7 DATA*tab* Team (2025). DATA*tab* online statistics calculator. DATAtab e.U. Graz, Austria. URL (online), https://datatab.net/ [accessed on 25 June 2025]

8 DiaGen. Hepatitis B Quantitative Rt-PCR kit (online), https://www.diagen.com.tr/hepatitis-b-quantitive-rt-pcr-kit [accessed on 19 February 2025].

9 COBAS TaqMan. HBV test for use with the high pure system (online), https://www.accessdata.fda.gov/cdrh_docs/pdf5/p050028c.pdf [accessed on 19 February 2025]

10 artus® HBV QS-RGQ Kit (online), file:///C:/Users/10006402/Downloads/artus-HBV-QS-RGQ-Kit-Performance-Characteristics-EN.pdf [accessed on 19 February 2025]

11 artus® HBV QS-RGQ Kit (online), file:///C:/Users/10006402/Downloads/artus-HBV-QS-RGQ-Kit-Performance-Characteristics-EN.pdf [accessed on 19 February 2025]
